# Patient, cured, victim or survivor of urological cancer? A qualitative study*

**DOI:** 10.1590/1518-8345.2715.3089

**Published:** 2018-11-29

**Authors:** Rafaela Azevedo Abrantes de Oliveira, Márcia Maria Fontão Zago

**Affiliations:** 1 Universidade Federal do Mato Grosso do Sul, Coxim, MS, Brazil.; 2 Universidade de São Paulo, Escola de Enfermagem de Ribeirão Preto, PAHO/WHO Collaborating Centre for Nursing Research Development, Ribeirão Preto, SP, Brazil.

**Keywords:** Cancer Survivor, Survivors, Anthropology Medical, Qualitative Research, Oncology Nursing, Narration

## Abstract

**Purpose:**

to describe the meanings that patients attribute to the term cancer survivor and to analyze the identities assumed by them according to their experience with the disease.

**Methods:**

qualitative study with a narrative method, theoretical framework of the medical anthropology and identity concept. The study included 14 participants, men and women, diagnosed with urologic cancer. The semi-structured interviews were performed at the individual’s home, after confirming participation.

**Results:**

eight participants assumed to be survivors, but five also assumed at least one other identity, in addition to cancer survivor. In contrast, among the six who defined themselves as cured, only one indicated another identity. Four considered themselves as victims and only two as cancer patients. However, the latter - cancer patient and victim - assumed at least one other associated identity.

**Conclusions:**

allowing patients to reflect on themselves and their experience with the disease, as well as attributing themselves a new identity, will be directly related to the wellbeing and momentum the survivor is going through. Therefore, it can direct care in the cancer survivorship phase according to each survivor’s individual context.

## Introduction

The concept of cancer survivor refers to someone that has gone through several changes and constant challenges with continuous difficulties, which can be positive and negative. According to this study, not all individuals would identify with the term survivor because they believe that it is not appropriate or does not define their experience^1^. The term cancer survivor for those diagnosed with cancer remains unclear and uncertain, and there is a lack of a consistent and usual operational and conceptual definition that hinders the suitability of the term among the public concerned, despite its widespread use^2^ ^-^ ^4^.

Research involving the identity of individuals with or after cancer shows that many respondents cannot define themselves as survivors, especially when they are still dealing with the disease. However, a qualitative descriptive study^5^ with 155 female African American breast cancer survivors showed that some of the women could define themselves as cancer survivors because they remained alive and would be survivors for the rest of their lives, reflecting that life is just starting over. In contrast, other women either did not believe that being a survivor reflected their history with the disease or thought the term was unfamiliar.

The human being is a biological entity inseparable from its culture, and cancer survivorship is a process constituted and lived by this being, i.e., it is a culturally variable process. However, a theoretical approach that focuses on the cultural and contextual aspects involved in the concept is necessary to obtain the meanings of cancer survivorship among cancer patients. The concern here is not only to write something, or highlight new terms (Cancer Survivors and Cancer Survivorship), but to mobilize patients, families, oncologists, nurses, researchers and others affected by the cancer experience^6^. It is from the knowledge of the survivors’ individuality, beliefs and values, and social dimensions that professionals can learn what is relevant to consider in the care of the cancer survivorship phase. Given the uncertainties, inconsistencies of the definition of being a cancer survivor, and the fact that it is not used in the Brazilian health context, the study sought to answer the following questions: How do cancer patients interpret the term cancer survivor? Which identities portray their experience with the disease? How is each identity interpreted? Thus, this study has two main objectives: to describe the meaning that the cancer patients attribute to the term cancer survivor, and to analyze the identities assumed by them according to their experience with the disease.

## Method

The cancer survivors are the “objects” of choice to study such phenomena and, assuming that their discourses are permeated by symbols, beliefs and cultural values, the medical anthropology theoretical reference was adopted, which will guide the analysis of the meanings based on the cancer survivors’ reports. Medical anthropology assumptions join the concepts of culture and disease and consist of deciphering the implicit and explicit meanings in the subjects’ language, interpreting their intentions, explanations, and historicity^7^.

The researchers assumed that the cancer patients’ perceptions of the health and disease process and their involvement during this process, besides the fluctuations of time and the changes resulting from living with cancer, will influence the construction and elaboration of the transition from being or feeling as a survivor, a patient, a victim or a cured individual, as discussed below^7^ ^-^ ^8^. In the anthropological approach, identity is like a sociocultural conditioned phenomenon, i.e., it refers to both collective identification and self-identification of individuals; they are correlated, considering that they are not innate, but they also cannot be detached from the individuals’ historical biography, their culture and their environment^8^.

The identity concept is very difficult to explain, considering its complexity and multi-dimensions, in addition to the diverse theoretical perspectives involved, including different explanatory terms. However, the basic meaning of identity refers to the place to which a person or group belongs and what is expressed as their self-image or common image that integrates them within themselves, or as part of a group, and also what differentiates them from others^8^.

It’s a qualitative exploratory study with a narrative method. Narratives are the main expressions used by individuals to tell their stories (i.e., dramas, defeats, achievements, joys). To narrate is the act of telling an event that has a beginning, middle and end, and allows accessing the other’s experience. This method is based on the premises^9^ ^)^ of individual narrative centered on experience. These premises refer to the act of narrating as a representation and reconstruction of events, time and place and experiences, emphasizing that they cannot be repeated exactly as they occurred because words never mean the same thing twice. The interest in construction and reconstruction in the narrative research guided by experience enables researchers to have a personal view of a narrative and that is treated as the only truth among many other narratives; and finally, the narrative serves as a transformation, which is the last premise and represents the personal changes that occurred throughout the individual’s experience with the disease.

The study participants are part of a larger project of concept analysis of cancer survivorship. A total of 14 participants, all diagnosed with urological cancer - bladder, kidney, prostate and testis - were selected according to the following inclusion criteria: adult patients (over 18 years old) diagnosed with urological cancer (regardless of the cancer type) and had completed primary treatment for at least three months; patients from both sexes, regardless of education level and socioeconomic status, who reported physical and psychological conditions for participation; and finally, patients residing within a 100 km from the city where the study took place.

After submission to the Research Ethics Committee and approval under protocol 503.385/2013, the participants were addressed at a São Paulo university hospital, where the researcher was present at the oncological urology outpatient clinic, where the follow-up appointments were conducted. Then, they were invited to participate in the study. Only one of them refused because the individual did not feel comfortable to participate. The others agreed to participate and signed the Terms of Free and Informed Consent. The total number of participants was determined by the quality of the interviews, which were finalized as soon as achieving objectives. Data were collected between October 2014 and November 2015.

Data collection technique included participant observation and a semi-structured interview which was addressed, recorded, and conducted following a script with the guiding questions: Who are you? Have you heard of cancer survivorship? What does being a survivor mean? Do you consider yourself a cancer survivor? More than one interview was necessary to guarantee access to in-depth narratives with detailed descriptions. There was an average of two interviews with each participant at their home; each interview lasted approximately 60 min. The names of the participants are fictitious and chosen by them, ensuring anonymity.

 Transcription was initiated by the main investigator after data collection, and the field diary was included in the text. The participants had the opportunity to read and revise the transcriptions, which helps to avoid wrong interpretations. Then, the inductive thematic analysis, a six-step process consisting of an interpretive analysis that searches for meanings, was initiated^10^ according to the common aspects, relationships and differences between them, expressed in subjects. The subject represents a level of meaning within the data body, regardless of its frequency, but dependent on the theoretical perspective of the researcher to interpret the results^10^. The process of the interview dynamics and the analyses of observations to understand how the participants perceived their disease experience was part of the construction of scientific knowledge through the hermeneutic circle. It is through this circle that the researcher describes, explains and interprets the narratives with understanding and alterity.

By examining the transcripts, relevant passages were highlighted and then analyzed as part of a whole to come to the understanding, explanation and interpretation of the common sense. The stories were grouped into a single and broad narrative synthesis, in the first person, involving the experience and transformations of all of the participants that were dialectically interpreted and discussed according to the category of choice. This synthesis was titled “What am I? Am I a cancer survivor? Self-reflection of identity after primary cancer treatment” and was used to discuss the thematic categories presented in the results, in which the excerpts from the participants’ speeches are marked in italics.

## Results

The study has 14 participants and the results were divided into two categories: I am not a patient, maybe a survivor or a victim, and I want to be cured; the meanings of the cancer survivor term. Table 1 shows the relevant clinical and social data of participants which complement the analysis. With a mean age of 60.8 years, the urological cancer group reported having at least one comorbidity associated with cancer, and only one participant had metastasis. The educational level was low, with functional illiteracy, and the participants were restricted to little writing and had difficulty interpreting texts.

**Table 1:** Sociodemographic and clinical characteristics of participants. Ribeirao Preto, SP, Brazil, 2015

Participants	Age*	Marital status	Education	Religion	Occupation	Family income	Type of cancer	Time since diagnosis*
P01	62	Married	High school	Catholic	Retired	3 MW^§^	Prostate	12 years
P02	68	Widowed	Junior high I^†^	Catholic	Housewife	1 MW^§^	Bladder	26 years
P03	62	Married	Junior high II^‡^	Deist	Retired	2 MW^§^	Bladder	5 years
P04	60	Married	Junior high II^‡^	Spiritualist	Doorman	2 MW^§^	Kidney (R^||^)	3 years
P05	60	Married	Junior high II^‡^	Catholic	Businesswoman	2 MW^§^	Kidney (L^¶^)	3 years
P06	62	Married	Junior high I^†^	Catholic	Retired	2 MW^§^	Prostate	3 years
P07	59	Married	Incomplete junior high I^†^	Evangelical	Trader	3 MW^§^	Bladder	2 years
P08	62	Married	Junior high II^‡^	Catholic	Lathe operator	3 MW^§^	Kidney (R^||^) and Bladder	9 years
P09	79	Married	Junior high I^†^	Spiritualist	Retired	2 MW^§^	Prostate	2 years
P10	63	Widowed	Junior high I^†^	Catholic	Retired	3 MW^§^	Prostate	3 years
P11	64	Married	Incomplete higher	Evangelical	Trader	3 MW^§^	Prostate	2 years
P12	56	Married	High school	Atheist	Retired	2 MW^§^	Kidney (L^¶^)	2 years
P13	54	Steady partner	High school	Catholic	Mechanic	2 MW^§^	Bladder	12 years
P14	41	Steady partner	Junior high I^†^	Evangelical	Clerk	2 MW^§^	Testicle (L^¶^)	3 years

*Until the end of the second interview (DEC/2015); †Junior High I - until fourth grade or fifth year; ‡Junior High II - until eight grade or ninth year; §MW - Minimum wage (around $280 American dollars); ||R- right; ¶L- left

**Figure 1:**
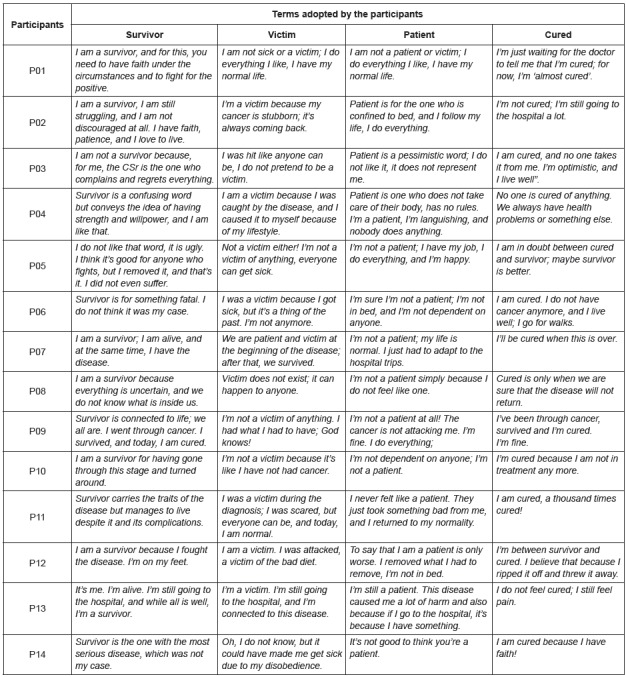
Characterization of the possible identities assumed by the study participants. Ribeirao Preto, SP, Brazil, 2015

Participants’ responses were diverse and reflected their emotional state at the time of the interviews. The expression ‘I do not know’ was mentioned quite often and was perhaps related to the fact that the authors put the participants under reflection on something that they had never stopped to think about, who am I? - which surrounded them with doubts. Participants went beyond the identity of being or not being a cancer survivor. In fact, the initial intention was not to discuss other terms, but they helped the participants rethink and organize their speeches about their identity. Therefore, we asked about being a patient or not, and being a victim of the disease or not, because the participants showed ambiguities and lack of clarity in the answers about being a cancer survivor or not. These identities helped the participants construct logical thinking patterns and to review their interpretations about being a cancer survivor, allowing them to assume one or more identities that represent them. Due to the diversity of the reflected and assumed identities, Figure 1 presents the synthesis of each participant’s speeches regarding these identities, in which the segments in bold emphasize the identities they assumed.

Eight participants reported being survivors, but five also assumed at least one other identity, in addition to cancer survivor. Among the six who defined themselves as cured, only one mentioned another identity. Four considered themselves as victims and only two as patients. However, the latter - patient and victim - assumed at least one other associated identity. This result shows that some individuals may adopt more than one identity to give meaning to their experience with the disease.

The words “patient” and “cured” make up the dyad that defines the health status of the patient in the Brazilian health biomedical context. In fact, in this context, there is no middle ground: either you are a patient or you are cured. The participant stressed this well: *here people only say I’m glad that you are cured! or What a pity you have cancer!* (P13). The moment the authors confront them with a new option - surviving the disease - they rethought the concept, there was a clash of ideas and conflicts with the health and disease context in which they are included, and new interpretations could be realized.

The disease is closely linked to the presence of signs and symptoms that express that something in the body is not well. Then, the search for treatment begins and, as long as the symptoms persist, the person will be or will feel like a patient. Therefore, the two participants who assumed the patient identity also reported they are or have been in a state of depression: *I still go to the hospital, I still feel pain. You may even be sick and not go to the doctor, but if you go it is because you’re feeling something. It’s how I feel about cancer. I have pain, and I go to the hospital, and therefore, I feel sick too* (P13). This participant reported the desire to commit suicide, which for him represented the most difficult moment of his life: *I tried to kill myself. The disease affected me very badly, and it was very difficult. I had a problem with my wife, I lost my job, I lost my mother and father, all at the same time* (P13).

The other participant who considered himself a patient and depressed takes sleeping pills and presents other health problems besides cancer, which aggravates his situation and increases the sense of uselessness: *I should not be sick, but I feel like I am and that matters because my life is not good. I’m full of health problems that doctors do not solve them. I just sleep* (P04). Assuming the patient identity expresses a state of overload, in which the body is weakened and the psychological domain is shaken, so that the negative characteristics of the word patient are exalted, resulting in emotional fatigue and low self-esteem.

Other participants preferred to minimize the disease and considered the word “patient” pessimistic: *I will not say that I am sick in my head because it only gets worse; I prefer to think that I am on the path to cure* (P12). In contrast, others considered themselves cured: *I am well, I finished the treatment, I have faith, I am optimistic, I am happy.* The cure is what every person wants to reach, even if they cannot. In this sense, the cured identity is the compensation that, at least from the social point of view, everything is fine and life remains aligned with the normal standards established by society. As outlined in Figure 1, it is easy to perceive the conviction of some participants that they will be cured with statements such as *when the pain disappears, when the doctor says,* and *when they do not need to go to the hospital*. The question of being cured or not is unique. Only one participant, the only one who experienced metastasis, was able to reflect on the cure from another perspective. For him: *Cure is when we are sure that the disease will not return, which is not the case of cancer, which can return, as it has returned* (P08).

Furthermore, the feelings of cure among those who do not share the uncertainty of the disease can come from the individual’s personal construction, desire and faith in being free from the disease as a reaction to a situation of incapability. It would be like assuming a compensatory logic, seeking to become resigned to a situation according to accepted morality and values, taking into account the normality of their past and the desire to reestablish it, together with sources of faith and hope, to achieve a healthy future with the cure of their disease. All of these feelings were expressed and justified by the participants, especially after reintegration into society: the *return to work, to daily activities, to normality*.

Those who identified as victims were associated with the idea of *not caring for the body, God allowed, I was attacked by the disease and have to live going to the hospital and know that “it”, the cancer, is still there*. These results reveal the persistent sense of guilt and concern that surrounds individuals who identify themselves as victims.

By analyzing why the eight participants had associated the survivor identity with their condition, the cancer was observed to be seen metaphorically as a war and, in that sense, if they are alive, it is because they won the battle. The idea of fight, conquest and victory is directly associated with being a cancer survivor: *I fight to survive* (P05), *I fight for the positive* (P01), *I cannot be discouraged, I have to fight always* (P02). One of the participants was able to synthesize this well: *I consider myself a winner simply because I am a destroyer of evils, I did not even feel the cancer and it was killing me. However, I managed to beat it. I’m a winner; it did not catch me, I caught it* (P04). Contextualized in this way, it is natural for the survivor to call himself a *destroyer of evils, victorious, winner,* or *warrior*. Cancer as a “war” can be understood as a conceptual metaphor - a battle in which there are victims and survivors. This metaphor explains why women with breast cancer are more likely to adopt the survivor identity after chemotherapy treatment, because it is a procedure that brings suffering and leaves marks^11^.

Another facet deduced from analysis of the narratives concerns the strong presence of religion in two different aspects: either between those who considered themselves to be survivors or those who used faith in God to feel cured: *To be a cancer survivor, you need faith in God and patience* (P02); *I am cured because I have faith in God* (P14). In Brazil, only a minority has no religion; most individuals are Catholic or Evangelical. Among the participants mentioned above, one is Catholic and the other Evangelical. There is no way to disregard the influence of religion and faith in the choice of identity, as the experience of the disease provides a moment of deep relationship with God and hope towards Him, that He will act and, in the end, everything will work out.

The fact that even after the primary treatment is completed the patients are still linked to the disease (performing tests, feeling pain and going to the hospital frequently) refers to the idea of survivorship. It is as if they are in a phase that soon will pass, but they know that they need to conduct the follow-up to ultimately attain the cure. The fact that they still look sick, even without feeling so, generates an ambiguous feeling, a liminality moment, of not feeling sick or cured, but rather a cancer survivor: *I’m living very well, but at the same time, I have the disease, I’m a cancer survivor* (P07); *A survivor carries the traits of the disease, but manages to live despite it and its complications* (P11); *I still go to the hospital a lot. I feel pain, I do a lot of exams, and I still take a lot of medication* (P02); *I’m alive, but I’m still going to the hospital, and while all is well, I’m a survivor* (P13).

Another issue concerns the possible reasons for not adopting the term cancer survivor - for judging it inappropriate and more suitable to those who have experienced a life-threatening event: *Survivorship is related to a person who has had a disease and almost died, who got rid of death. I did not get to that point. I was scared and worried when I heard my diagnosis, but not life-threatening* (P11); *Survivor is for someone who has gone through a fatal, risky situation* (P04); *Survivor, in my head, I am not, because it was not fatal* (P06)*; Survivor, to me, is the one with a very serious disease and then gets cured and survives, and I think that the cancer is only very serious depending on the situation; mine was not, I think it wasn’t serious because I found out early, in time for curing* (P14)*; Survivor, in my understanding, would be more for accidents, you survived a car accident and you’re alive* (P02).

Applying the concept of survivorship to people with cancer is something relatively new among Brazilians. It is a new language and, therefore, it is natural that the participants still associate it with serious or very serious contexts, such as accidents in general. Only one participant who considered his disease serious assumed the cancer survivor identity: *Surviving is related to living after a serious episode, and I arrived really badly here at the hospital; my case was serious, and I’m alive!* (P13). Other aspects that may influence this association are the type of cancer and the treatments to which people are subjected.

There is a clear lack of studies on the topic among cancer survivors in developing countries. This lack of research expresses the lack of evidence or even the little interest in discussing the topic in these regions, which interferes with the construction of the cancer survivor identity by those who do not know the term. In their speeches, several participants reported never having thought about *identity, who they are or about being a cancer survivor*. They reaffirmed the need and importance of their oncologists’ opinion: *This issue of seeing with one identity depends very much on how the doctor treats us, what he or she tells us. I think I could see survival differently if someone explained it to me better* (P05); *The doctor said that if I removed the prostate, I would be cured, and I removed it, so that’s how I feel* (P11); *I’ll wait to see what the doctors have to say* (P12); *the doctors tell me whether I’m well or not, and they have not said anything bad; they cheer me when I go there. It encourages me to feel cured* (P14).

The physician’s opinion is culturally strong, and the biomedical model praises this role of healers or curing agents in front of patients. Trust in this professional is established by his direct relationship with the patient and is based on the social recognition that he has superior knowledge: *I do not know what I am; I have to ask my doctor, he knows more* (P01). This situation arises from the fact that the doctors have knowledge about the disease, along with the technological resources to see what cannot be seen by the patients; thus, they can define and employ the best therapy for a much-desired normal life.

## Discussion

This research focused on patients with urologic cancer, a specific group with a severity level lower than other more devastating cancers. To date, no studies involving this group are known, except studies with individuals diagnosed with prostate cancer. The methodology discussed allows researchers to access the individuals’ narratives and let their stories flow, without forcing or inducing them to get a result. In the inductive analysis, there was no association between the participants’ sociodemographic characteristics and the type of identity adopted. However, each participant has a narration for his/her experience with the disease, and it contains personal, individual, cultural, social, and economic aspects of his/her life; the clinical aspects make the way they deal with disease, face it and see themselves unique.

Although the concepts of cancer survivorship and cancer survivor have already been discussed in some countries, they are unknown in others, although there are large numbers of survivors of the disease in developing countries. Because it is not approached by health professionals and researchers, when the term is put to the cancer patient as a question, it is reflected and interpreted according to their contexts and values, and they are dialectically confronted with the experience they lived, noting that what is not similar to the conventional is often ignored, misinterpreted, being criticized and discriminated.

The results demonstrated the interpretation of a group of cancer survivors regarding a series of terms - patient, cured, victim and cancer survivor. The point is not to assume a single identity that is representative of each one’s experience, but that in narrating their stories the patients oscillate between a set of emotions that are positive or negative over time, and that directly interferes with their interpretation of who they really are and what identities define them; it is natural that they recognize more than one identity in a single moment or change them over time, in a process of construction and gaining knowledge about themselves. There is a specific time for patients to recognize themselves as victims or patients, usually at the beginning; these terms are less incorporated after a year of follow-up and consequent improvement^12^. 

Identity oscillates between different cultural groups; it is neither fixed nor universal. Through shared symbols and beliefs, feeling as a cancer survivor, as cured, as patient or victim may or may not coincide with being recognized as such, implying fluctuations in the experience of this condition, sometimes being convenient, sometimes inconvenient, sometimes adequate or inadequate to what is being experienced by the individual^13^. Therefore, from the point of view of anthropology, the identity is never given to someone, but it is always constructed through elements present in each cultural group^8^.

Following this logical thinking, it is easy to understand why extremely ill and cured are the terms most understood by the patient. Cured refers to what is good, to being healthy, regardless of the course of the disease, considering that only one patient experienced recurrence, and reflects the possible absence of cure in face of the possibility of its recurrence. The belief that one day they may indeed be free from the disease is very strong and overcomes the uncertainties that the disease conveys. In one study^14^, the authors addressed the false expectations of cure created by the patient because the cancer cure is related to survivorship statistics and evaluation protocols; the cure is achieved when the patient’s survival reaches a pre-established mark in years. However, this benchmark should not be the criterion for considering that a patient is cured, because such a state can never be affirmed with absolute certainty in oncology, as the patient’s environment is permeated by uncertainties, even in relation to their own possibility of cure.

The term cancer survivor was interpreted as a “period of struggle” because as long as the patients are struggling for the cure, they are survivors; however, the final stage is always the cure, and all of these feelings are managed by faith. Religion is part of the Brazilian culture and influences the construction of their identity and how to deal with serious illness. Thus, during cancer survivorship the participants, with the support of religion/spirituality, tend to develop a sense of hope and satisfaction with life and, consequently, have lower levels of depression. Hence, religion/spirituality is recognized as a bargaining strategy for seeking the strength to survive, fight, be a cancer survivor and achieve the cure.

Considering oneself as a cancer survivor has positive effects on the quality of life and physical and mental well-being, which are directly proportional to the positive aspects of identity, self-esteem and autonomy and indirectly proportional to negative stigma issues, especially those attributed to cancer. The presence of high self-esteem induces individuals to consider themselves as survivors of the disease^15^ ^-^ ^16^. Thus, the cancer survivor identity may be associated with psychological well-being^5^ ^,^ ^16^ and personal growth^4^ ^,^ ^15^ ^-^ ^17^. Another point is that patients who tend to consider themselves as survivors have a deep desire to help others who experience the same situation^18^. They also wish to participate in related activities, such as support groups and social events^2^ ^,^ ^16^.

Being a cancer survivor, from the perspective of Brazilians diagnosed with urological cancer, is related to the fact that they still bear “traces” of the disease, but to also have faith and willpower and to always fight for life, not to become discouraged but to trust that everything will go well and live, regardless of whether you are frequently at the hospital, if you feel pain or if you use medications. Thus, before so many meanings, it is possible to say that the identity of being or feeling like a cancer survivor requires a genuine experience with this condition so that the patient can attribute more reliable meanings to the phenomenon, accepting or refusing it, strengthening or weakening the feelings of belonging to it^13^. 

The literature has already shown that the patient’s psychological aspects have an influence on their assumed identity^15^ ^-^ ^16^. Many studies have also associated potential post-cancer identities with not only one’s experience with the disease, but also mental aspects and physical well-being. Accompanying and recognizing, the individual’s psychological well-being and how patients cope with their disease through self-definition helps health care professionals to manage and improve their care.

 Where the culture to identify the disease and seek cure prevails, it is extremely difficult to insert a new term, in this cancer survivor, although not impossible because it is a concept more appropriate to the group in question. However, the consolidation of a new term requires a great deal of effort by the professionals, patients and family members; moreover, effective public policies are necessary to socially introduce a different perspective of seeing cancer, which gives patients a new way of seeing themselves^14^. The doctor-patient and nurse-patient relationships are key pieces in the introduction of a new term, and the consequent re-education and subsequent construction of a new identity. Language is the main resource to facilitate and promote the understanding and interpretation of the disease by individuals, because it can reduce the natural uncertainties inherent to the disease. Thus, the language used by health professionals during the survivorship period interferes with the way patients see their identity, because the way people talk and communicate about the disease can influence their readjustments to daily life and the quality of their experience as a cancer survivor^19^.

Before the discussion about the change of identities and what would better represent those who were diagnosed with cancer and concluded the treatment, this study fortifies the researches in this field for being a forerunner among patients with urological cancer, besides contributing to the understanding about the endorsement of several identities, consolidating the concept of cancer survivorship and cancer survivor. Furthermore, it is important to enlarge the investigation to patients with different clinical and social profiles. The limitations are restrained to patients with urologic cancer diagnosis and do not consider the gender, age, disease stage, and other variables, which could influence the patients’ interpretation about themselves and, consequently, in the endorsement of a new identity.

## Conclusion

This study concludes that allowing patients to reflect on themselves and their experiences with the disease, as well as to attribute themselves a new identity, will be directly related to the well being and momentum that the survivor is going through. Therefore, it can direct care in the cancer survivorship phase according to each survivor’s individual context. The issue is not in standardization, inserting the affected individuals into a group or creating labels, or even importing what does not culturally reside in the social contexts of other regions. The issue is in the assumption of a new, relevant language, a matter of public and social health that refers to a heterogeneous group that has special needs and demands special care. What really matters is to recognize how these individuals live and thrive after primary treatment, how they cope with the disease and how to improve follow-up strategies to manage the health promotion of a growing group of individuals who live and will live with the disease, associating the identity issue with a real characteristic of cancer - the absence of definite cure, despite the guarantee of maintaining the quality of life. Lastly, to coordinate patient-centered care, it is important to demystify beliefs, strengthen new standards and concepts, and deepen the research studies that foster the expansion of knowledge of this group.
